# Crystal engineering of a 1:1 5-fluoro­cytosine–4-hy­droxy­benzaldehyde cocrystal: insights from X-ray crystallography and Hirshfeld analysis

**DOI:** 10.1107/S2056989025004463

**Published:** 2025-05-23

**Authors:** Marimuthu Sangavi, Marimuthu Mohana, Ray J. Butcher, Colin D. McMillen

**Affiliations:** a Department of Chemistry Thanthai Periyar Government Arts and Science College Tiruchirappalli-620 023 (Affiliated to Bharathidasan University, Tiruchirappalli 620 024 Tamil Nadu India) Tamil Nadu India; b Department of Chemistry, Periyar Maniammai Institute of Science and Technology (Deemed to be University), Thanjavur 613 403, Tamil Nadu, India; c Department of Chemistry Howard University, Washington, DC 20059 USA; d Department of Chemistry, Clemson University, H.L. Hunter Laboratories, Clemson, SC 29634, USA; Harvard University, USA

**Keywords:** cocrystal, supra­molecular network, dimeric motif, tetra­meric motif, Hirshfeld surface analysis,fingerprint plots

## Abstract

The 1:1 cocrystal of 5-fluoro­cytosine (5FC) and 4-hy­droxy­benzaldehyde (4HB), C_4_H_4_FN_3_O·C_7_H_6_O_2_ has been synthesized and its structure characterized by single-crystal X-ray diffraction and Hirshfeld surface analysis. The compound crystallizes in the monoclinic *P*2_1_/*c* space group. A robust supra­molecular architecture is stabilized by N—H⋯O, N—H⋯N, C—H⋯O and C—H⋯F hydrogen bonds, forming *R*_2_^2^(8), *R*_4_^4^(22), *R*_6_^6^(32), and *R*_8_^8^(34) ring motifs. The N—H⋯O and N—H⋯N hydrogen bonds form strong directional inter­actions, contributing to the *R*_2_^2^(8) and *R*_8_^8^(34) motifs through dimeric and extended ring structures. O—H⋯O inter­actions link 5FC and 4HB mol­ecules, generating an *R*_6_^6^(32) ring that enhances the packing. Weaker C—H⋯F bonds help form the *R*_4_^4^(22) tetra­meric motif, supporting the overall three-dimensional supra­molecular framework. Additionally, C—F⋯π inter­actions between the fluorine atom and the aromatic ring add further to the crystal cohesion. Hirshfeld surface analysis and two-dimensional fingerprint plots confirm that O⋯H/H⋯O contacts are the most significant, highlighting the central role of hydrogen bonding in the stability and organization of the crystal structure.

## Abstract 2

The cocrystal of 5-fluoro­cytosine and 4-hy­droxy­benzaldehyde (1/1), C_4_H_4_FN_3_O·C_7_H_6_O_2_, crystallizes in the monoclinic *P*2_1_/*c* space group. The crystal structure features a robust supra­molecular network stabilized by N—H⋯O, N—H⋯N, O—H⋯O, C—H⋯O and C–H⋯F hydrogen bonds, forming diverse ring motifs including *R*_2_^2^(8), *R*_4_^4^(22), *R*_6_^6^(32), and *R*_8_^8^(34). Additional C—F⋯π inter­actions contribute to the crystal cohesion. Hirshfeld surface analysis reveals that O⋯H/H⋯O contacts dominate the inter­molecular inter­actions, emphasizing the key role of hydrogen bonding in the crystal packing.

## 1. Chemical context

Cocrystals have gained considerable attention in supra­molecular chemistry for their ability to improve the physical and chemical properties of active pharmaceutical ingredients (APIs) and functional materials without altering the mol­ecular structure of the drug. They are defined as crystalline, single-phase solids composed of two or more distinct mol­ecular and/or ionic compounds, typically in a stoichiometric ratio, which are neither simple salts nor solvates (Aitipamula *et al.*, 2012 ▸; Almarsson & Zaworotko, 2004 ▸). Cocrystals are stabilized through non-covalent inter­actions such as hydrogen bonding, π–π stacking, halogen bonding, and van der Waals forces. Their design is guided by the principles of crystal engineering, involving the careful selection of suitable coformers and the application of supra­molecular synthons, such as the *R_2_*^2^(8) hydrogen-bonded motif (Etter, 1990 ▸; Etter *et al.*, 1990 ▸; Desiraju, 1995 ▸). In the pharmaceutical industry, cocrystallization offers a promising strategy for enhancing the solubility, stability, and bioavailability of poorly soluble drugs. (Alvani & Shayanfar, 2022 ▸; Shi *et al.*, 2024 ▸). Compared to conventional techniques such as salt formation, micronization, solid dispersion, amorphous forms, and encapsulation, cocrystals offer the advantage of maintaining a stable crystalline structure, which facilitates detailed characterization by X-ray diffraction (Bolla & Nangia, 2016 ▸; Bolla *et al.*, 2022 ▸).
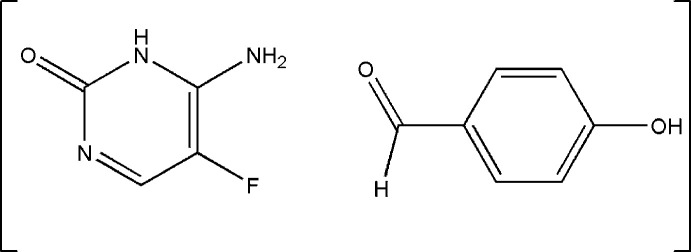


## 2. Structural commentary

**Figure 1:**
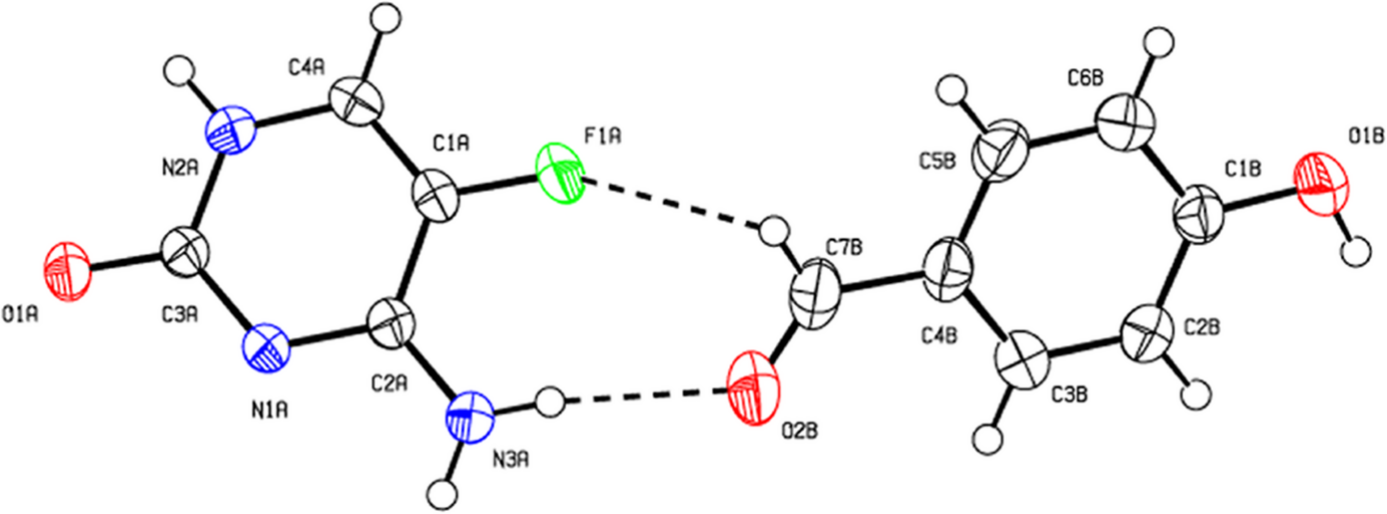
The mol­ecular structure of the title cocrystal with displacement ellipsoids drawn at the 50% probability level. Hydrogen bonds are shown as dashed lines.

Single-crystal X-ray diffraction analysis reveals that the title compound crystallizes in the monoclinic *P*2_1_/*c* space group with one mol­ecule each of 5-fluoro­cytosine (5FC) and 4-hy­droxy­benzaldehyde (4HB) present in the asymmetric unit. An ellipsoid plot of the compound is shown in Fig. 1 ▸. Proton transfer does not occur between the hydroxyl group of benzaldehyde and the pyrimidine ring nitro­gen atom of 5FC. The C—O bond length in the hydroxyl group of the 4HB mol­ecule is 1.3520 (13) Å, with the corresponding inter­nal bond angle [C2*A*—N1*A*—C3*A* = 120.00 (8)°] in agreement with reported literature values (Louis *et al.*, 1982 ▸; Mohana *et al.*, 2016 ▸, 2023 ▸; Sangavi *et al.*, 2024 ▸).

## 3. Supra­molecular features and Hirshfeld surface analysis

**Figure 2:**
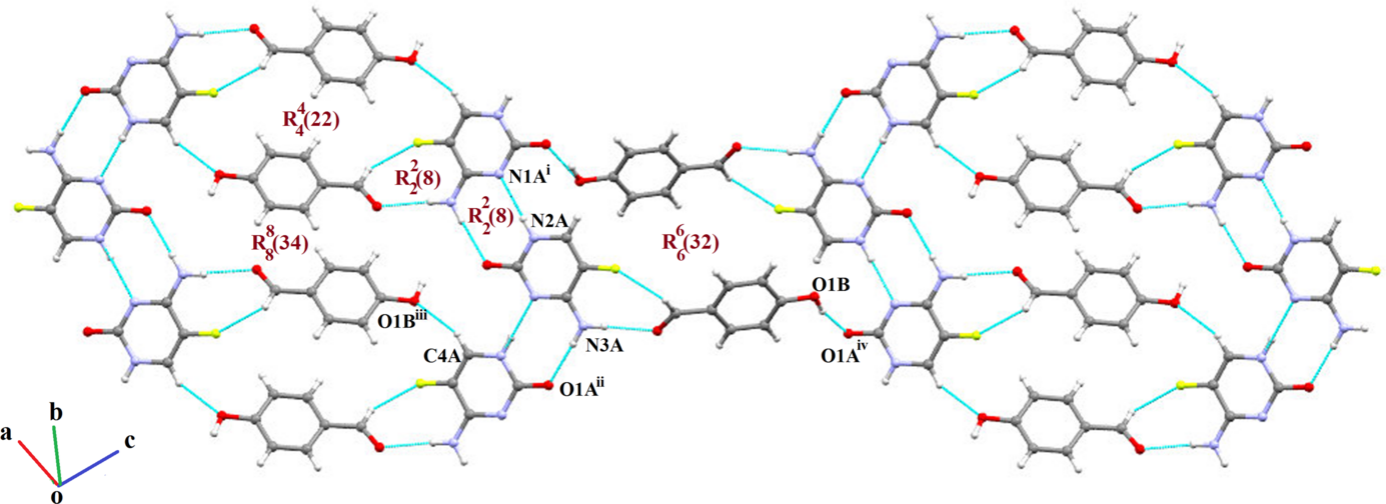
Three-dimensional supra­molecular cage-like architecture formed *via* N—H⋯O, N—H⋯N, O—H⋯O, C—H⋯F and C—H⋯O hydrogen bonds. [Symmetry codes: (i) −*x*, *y* + 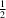, −*z* + 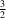; (ii) −*x*, *y* − 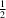, −*z* + 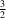; (iii) −*x* + 2, −*y* + 1, −*z* + 1; (iv) *x* + 1, −*y* + 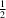, *z* − 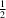.]

**Figure 3:**
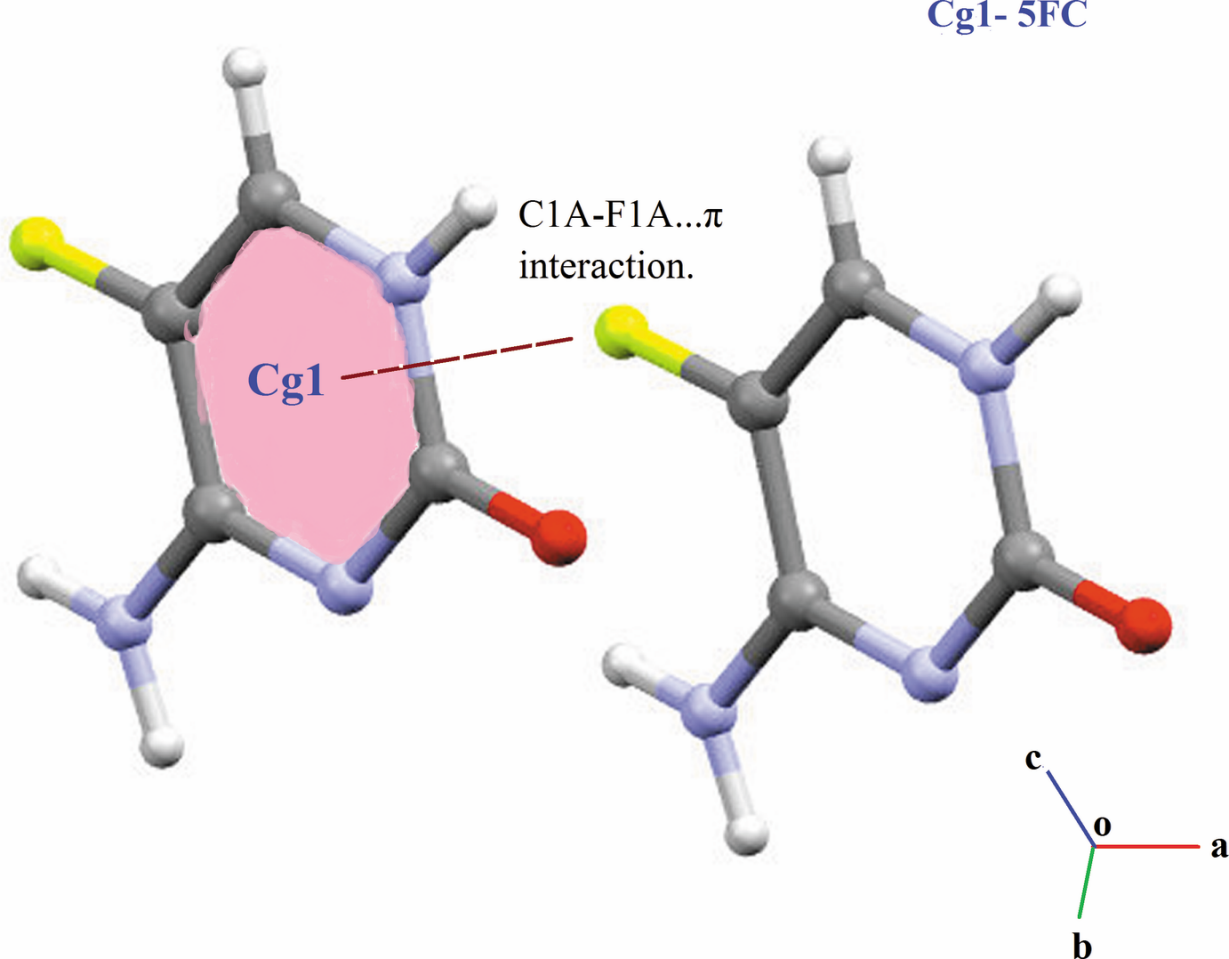
A view of the C—F⋯π inter­action (symmetry operation 1 + *x*, *y*, *z*).

The primary inter­action motif is formed *via* N—H⋯O and C—H⋯F hydrogen bonds (Table 1 ▸). The N4*A* amino group and F1*A* atom of the 5FC mol­ecule inter­act with the O2*B* and C7*B* atoms of the 4HB mol­ecule, resulting in an 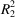
(8) heterodimeric synthon. Heterodimers are further linked through a weak C—H⋯O^iii^ [symmetry code: (iii) −*x* + 2, −*y* + 1, −*z* + 1] hydrogen bond involving the C4*A* atom of 5FC and the O1*B* atom of 4HB. The inter­action leads to the formation of an 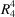
(22) tetra­meric synthon. The tetra­meric motif is further extended through a homodimeric 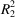
(8) synthon, formed by N—H⋯N^i^ [symmetry code: (i) −*x*, *y* + 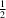, −*z* + 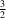] and N—H⋯O^ii^ [symmetry code: (ii) −*x*, *y* − 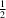, −*z* + 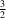] hydrogen bonds. These inter­actions involve atoms N1*A*, N2*A*, N3*A* and O1*A* of the 5-fluoro­cytosine (5FC) mol­ecule. The formation of this homodimeric synthon bridges adjacent tetra­meric units, resulting in a large 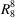
(34) ring motif. The alternating arrangement of 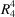
(22) and 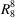
(34) rings leads to the development of a three-dimensional supra­molecular cage-like architecture. This network is further consolidated by O—H⋯O hydrogen-bonding inter­actions between the O1*A* atom of the 5FC mol­ecule and the hydroxyl (–OH) group of the 4-hy­droxy­benzaldehyde (4HB) mol­ecule. The hydrogen bonding occurs *via* an O—H⋯O^iv^ [symmetry code: (iv) *x* + 1, −*y* + 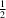, *z* − 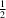] inter­action, forming an 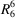
(32) ring motif (Fig. 2 ▸). This inter­action strengthens the packing and adds complexity to the supra­molecular network. In addition to hydrogen bonding, the crystal structure is further consolidated by weak C—H⋯F and C—F⋯π inter­actions. The C—F⋯π inter­action (Fig. 3 ▸) is observed between 5FC mol­ecules [C1*A*⋯*Cg*^v^ = 3.2676 (9) Å, C1*A*—F1*A*⋯*Cg* = 89.41 (6)°, where *Cg* is the centroid of the 5FC ring; symmetry code: (v) 1 + *x*, *y*, *z*]. The observed angle is consistent with values reported in the literature (Sikorski *et al.*, 2005 ▸; Vangala *et al.*, 2002 ▸).

**Figure 4:**
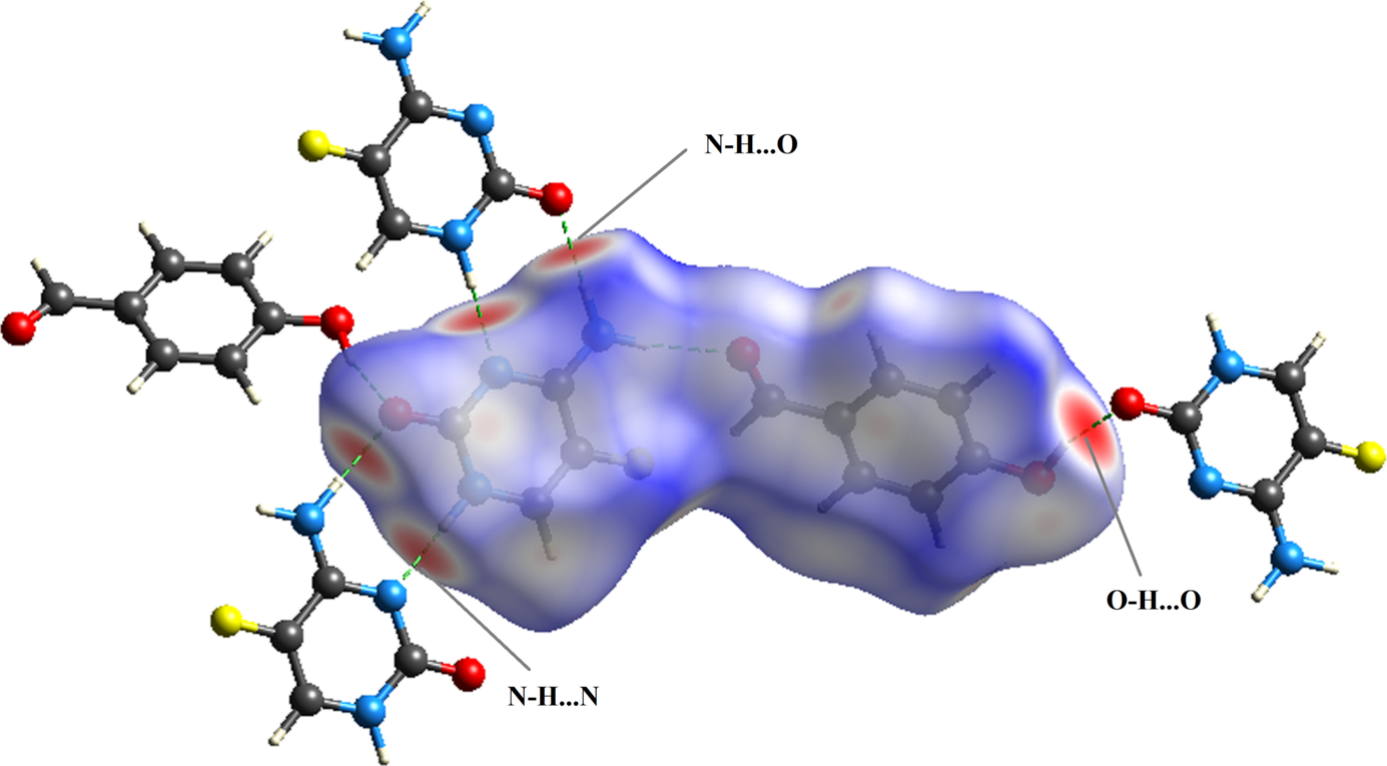
The Hirshfeld surface mapped over *d*_norm_ showing the N—H⋯O, N—H⋯N and O—H⋯O inter­actions as dashed gray lines.

Hirshfeld surface (HS) analysis was performed for the title compound to visualize and qu­antify its inter­molecular inter­actions. Fig. 4 ▸ presents the van der Waals inter­actions using a Hirshfeld surface mapped over *d*_norm_ (Spackman & Jayatilaka, 2009 ▸), generated with *Crystal Explorer 21* (Spackman *et al.*, 2021 ▸). This analysis reveals significant inter­molecular hydrogen bonds of the types N—H⋯O, N—H⋯N and O—H⋯O inter­actions. In the surface representation, red areas indicate strong hydrogen bonding, blue regions correspond to contacts close to the sum of the van der Waals radii, and white regions represent weaker inter­actions.

**Figure 5:**
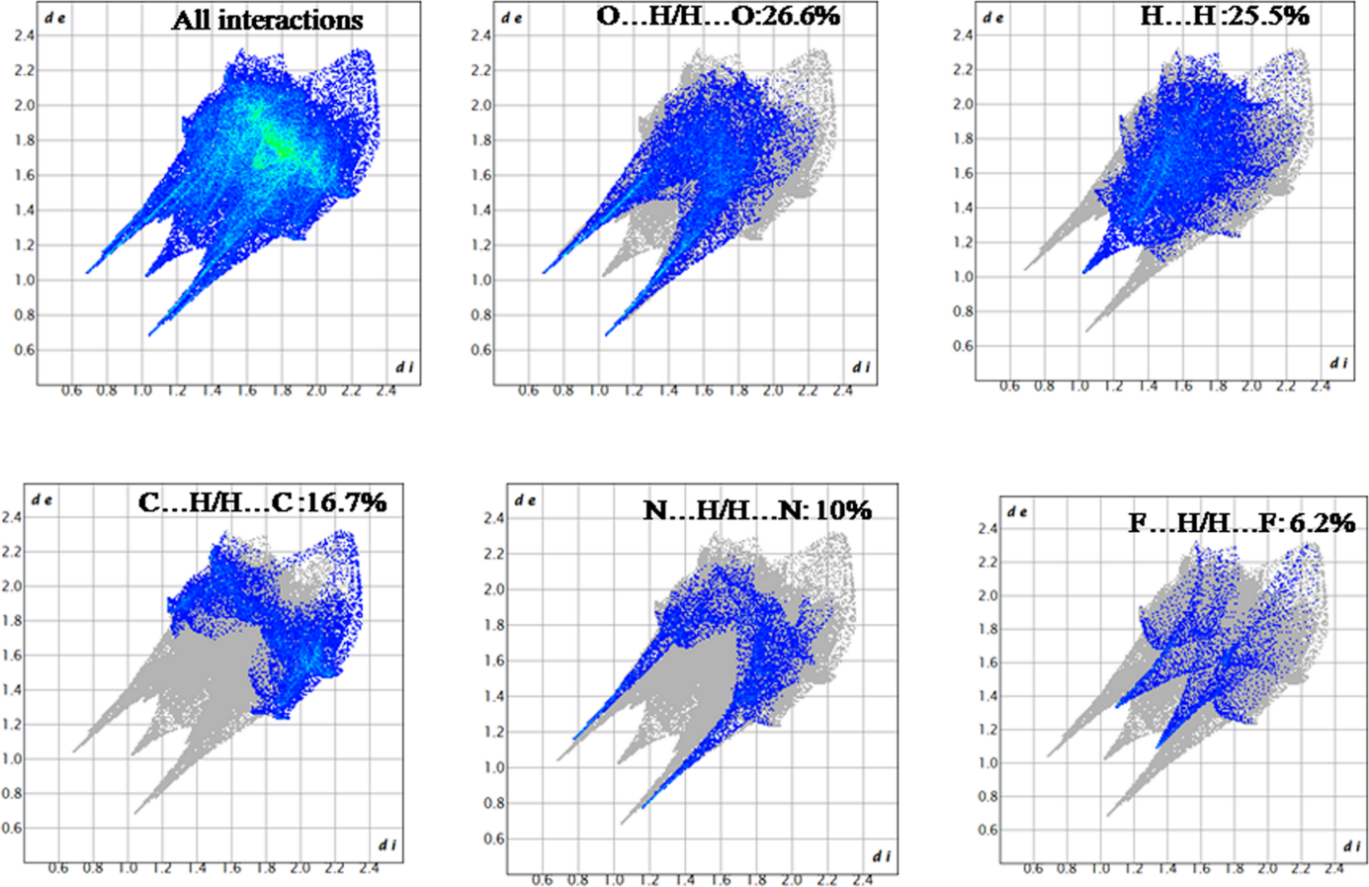
Fingerprint plots showing the total contribution of individual inter­actions and those delineated into O⋯H/H⋯O, H⋯H, C⋯H/H⋯C, N⋯H/H⋯N and F⋯H/H⋯F inter­actions.

To analyze the relative contributions of different inter­molecular inter­actions, two-dimensional fingerprint plots were generated (McKinnon *et al.*, 2007 ▸) and these are shown in Fig. 5 ▸. These plots indicate that the most prominent contacts are O⋯H/H⋯O (26.6%), followed by H⋯H (25.5%), C⋯H/H⋯C (16.7%), N⋯H/H⋯N (10.0%) and F⋯H/H⋯F (6.2%). The crystallographic analysis reveals a robust supra­molecular network in the title compound, stabilized by hydrogen bonds (N—H⋯O, N—H⋯N, O—H⋯O and C—H⋯F) and C—F⋯π inter­actions, forming a three-dimensional cage-like supra­molecular architecture. Hirshfeld surface analysis highlights prominent O⋯H/H⋯O inter­actions, alongside other significant contacts, contributing to crystal stability. The study demonstrates how non-covalent inter­actions, including hydrogen-bonding and π inter­actions, govern the mol­ecular packing and cohesion, supporting the principles of supra­molecular chemistry in crystal engineering.

## 4. Database survey

5-Fluoro­cytosine (5FC) is a synthetic anti­mycotic compound, first synthesized in 1957 and widely used as an anti­tumor agent. It is also active against fungal infection (Portalone & Colapietro, 2007 ▸; Vermes *et al.*, 2000 ▸). It becomes active by deamination of 5FC into 5-fluoro­uracil by the enzyme cytosine deaminase (CD) and inhibits RNA and DNA synthesis (Morschhauser, 2003 ▸). The Cambridge Structural Database (CSD, v5.45, June 2024; Groom *et al*., 2016 ▸) reference codes for the monohydrate are BIRMEU, BIRMEU01, BIRMEU02, BIRMEU03, MEBQUG, MEBQIU, MEBQOA and GATMUL (Louis *et al.*, 1982 ▸; Portalone & Colapietro, 2006 ▸; Hulme & Tocher, 2006 ▸; Portalone, 2011 ▸), and for the polymorphs: DUKWIQ, DUKWAI and DUKWEM (Tutughamiarso *et al.*, 2009 ▸). A wide range of cocrystals has also been documented, such as XOQQUS, MECTUL, MECVEX, MECVIB, MECVOH, MECVUN, MECWAU, MECWEY, MECWOI, MECWUO, MECXEZ, MECXID, MECXOJ, GIFWIF, UJUJAM, and POCWUD (Souza *et al.*, 2019 ▸;Tutughamiarso *et al.*, 2012 ▸; Tutughamiarso & Egert, 2012 ▸; Mohana *et al.*, 2016 ▸, 2023 ▸; Sangavi *et al.*, 2024 ▸). Salts include WEWZAA01, SIJXAM, SIJXIU, SIJXUG, EDATOS, GIFWEB, POCXAK, ZAPFEE and ROLTUJ WEWZAA01, SIJXAM, SIJXIU, SIJXUG, EDATOS, GIFWEB, POCXAK, ZAPFEE and ROLTUJ (Perumalla *et al.*, 2013*a* ▸,*b* ▸; Prabakaran *et al.*, 2001 ▸; Mohana *et al.*, 2017 ▸; Karthikeyan *et al.*, 2014 ▸) have been reported in the literature. 4-Hy­droxy­benzaldehydes are potential therapeutic agents for the treatment of human angiostrongyliasis. The crystal structure of 4-hy­droxy­benzaldehyde (Jasinski *et al.*, 2008 ▸), as well as its cocrystal (Nowak & Sikorski, 2023 ▸) and polymorphic forms (Simões *et al.*, 2013 ▸) have also been reported. 5FC contains multiple hydrogen-bond donors and acceptors, including amino and carbonyl groups, and 4-HBA offers hydroxyl and aldehyde functionalities capable of forming hydrogen bonds, along with an aromatic ring that can engage in π–π inter­actions. The present work focuses on the supra­molecular hydrogen bonding inter­actions in the crystal structure of 1:1 cocrystals of 5-fluoro­cytosine-4-hy­droxy­benzaldehyde.

## 5. Synthesis and crystallization

The title compound was synthesized by mixing a hot ethano­lic solution of 5-fluoro­cytosine with 4-hy­droxy­benzaldehyde in a 1:1 molar ratio. The solution was heated in a water bath at 333 K for 30 minutes and then allowed to cool slowly to room temperature. After a few days, colorless crystals had separated out of the mother liquor.

## 6. Refinement

Crystal data, data collection and structure refinement details are summarized in Table 2 ▸. The H atoms of the N—H, –NH_2_ and OH groups were located in difference-Fourier maps and refined freely. Other H atoms were placed geometrically (C—H = 0.93 Å) and refined using a riding model with *U*_iso_(H) = 1.2*U*_eq_(C).

## Supplementary Material

Crystal structure: contains datablock(s) I. DOI: 10.1107/S2056989025004463/oi2017sup1.cif

Structure factors: contains datablock(s) I. DOI: 10.1107/S2056989025004463/oi2017Isup2.hkl

Supporting information file. DOI: 10.1107/S2056989025004463/oi2017Isup3.cml

CCDC reference: 2452037

Additional supporting information:  crystallographic information; 3D view; checkCIF report

## Figures and Tables

**Table 1:** Hydrogen-bond geometry (Å, °)

*D*—H⋯*A*	*D*—H	H⋯*A*	*D*⋯*A*	*D*—H⋯*A*
N2*A*—H1⋯N1*A*^i^	0.88 (1)	2.06 (1)	2.9354 (12)	175 (1)
N3*A*—H1*CC*⋯O2*B*	0.89 (1)	2.10 (1)	2.9848 (13)	170 (1)
N3*A*—H1*A*⋯O1*A*^ii^	0.90 (1)	2.04 (1)	2.9328 (12)	176 (1)
C4*A*—H4*A*⋯O1*B*^iii^	0.93	2.48	3.2905 (14)	145
O1*B*—H1*B*⋯O1*A*^iv^	0.86 (2)	1.85 (2)	2.6934 (13)	166 (2)
C6*B*—H6*B*⋯F1*A*^iii^	0.93	2.56	3.3446 (14)	143
C7*B*—H7*B*⋯F1*A*	0.93	2.51	2.9886 (14)	112

Symmetry codes: (i) 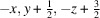; (ii) 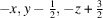; (iii) 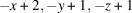; (iv) 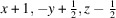.

**Table 2:** Experimental details

Crystal data	
Chemical formula	C_4_H_4_FN_3_O·C_7_H_6_O_2_
*M*_r_	251.22
Crystal system, space group	Monoclinic, *P*2_1_/*c*
Temperature (K)	297
*a*, *b*, *c* (Å)	4.2126 (1), 9.6687 (1), 26.8628 (5)
β (°)	94.186 (1)
*V* (Å^3^)	1091.21 (3)
*Z*	4
Radiation type	Cu *K*α
μ (mm^−1^)	1.07
Crystal size (mm)	0.27 × 0.21 × 0.17

Data collection	
Diffractometer	XtaLAB Synergy, Dualflex, HyPix
Absorption correction	Analytical (*CrysAlis PRO*; Rigaku OD, 2023 ▸)
*T*_min_, *T*_max_	0.782, 0.840
No. of measured, independent and observed [*I* > 2σ(*I*)] reflections	19824, 2243, 2127
*R*_int_	0.019
(sin θ/λ)_max_ (Å^−1^)	0.630

Refinement	
*R*[*F*^2^ > 2σ(*F*^2^)], *wR*(*F*^2^), *S*	0.034, 0.101, 1.04
No. of reflections	2243
No. of parameters	180
No. of restraints	4
H-atom treatment	H atoms treated by a mixture of independent and constrained refinement
Δρ_max_, Δρ_min_ (e Å^−3^)	0.20, −0.19

Computer programs: *CrysAlis PRO* (Rigaku OD, 2023 ▸), *SHELXT* (Sheldrick, 2015*a* ▸), *SHELXL2019/2* (Sheldrick, 2015*b* ▸), *PLATON* (Spek, 2020 ▸), *Mercury* (Macrae *et al.*, 2020 ▸), *POVRay* (Cason, 2004 ▸) and *publCIF* (Westrip, 2010 ▸).

## supplementary crystallographic information

### 4-Amino-5-fluoro-1H-pyrimidin-2-one–4-hydroxybenzaldehyde (1/1) Crystal data

**Table:**

C_4_H_4_FN_3_O·C_7_H_6_O_2_	*F*(000) = 520
*M**_r_* = 251.22	*D*_x_ = 1.529 Mg m^−^^3^
Monoclinic, *P*2_1_/*c*	Cu *K*α radiation, λ = 1.54184 Å
*a* = 4.2126 (1) Å	Cell parameters from 18043 reflections
*b* = 9.6687 (1) Å	θ = 3.3–76.2°
*c* = 26.8628 (5) Å	µ = 1.07 mm^−^^1^
β = 94.186 (1)°	*T* = 297 K
*V* = 1091.21 (3) Å^3^	Block, colorless
*Z* = 4	0.27 × 0.21 × 0.17 mm

### 4-Amino-5-fluoro-1H-pyrimidin-2-one–4-hydroxybenzaldehyde (1/1) Data collection

**Table:**

XtaLAB Synergy, Dualflex, HyPix diffractometer	2243 independent reflections
Radiation source: micro-focus sealed X-ray tube	2127 reflections with *I* > 2σ(*I*)
Detector resolution: 10.0000 pixels mm^-1^	*R*_int_ = 0.019
ω scans	θ_max_ = 76.2°, θ_min_ = 3.3°
Absorption correction: analytical (CrysAlisPro; Rigaku OD, 2023)	*h* = −3→5
*T*_min_ = 0.782, *T*_max_ = 0.840	*k* = −12→12
19824 measured reflections	*l* = −33→33

### 4-Amino-5-fluoro-1H-pyrimidin-2-one–4-hydroxybenzaldehyde (1/1) Refinement

**Table:**

Refinement on *F*^2^	Secondary atom site location: difference Fourier map
Least-squares matrix: full	Hydrogen site location: mixed
*R*[*F*^2^ > 2σ(*F*^2^)] = 0.034	H atoms treated by a mixture of independent and constrained refinement
*wR*(*F*^2^) = 0.101	*w* = 1/[σ^2^(*F*_o_^2^) + (0.0563*P*)^2^ + 0.2241*P*] where *P* = (*F*_o_^2^ + 2*F*_c_^2^)/3
*S* = 1.04	(Δ/σ)_max_ = 0.001
2243 reflections	Δρ_max_ = 0.20 e Å^−^^3^
180 parameters	Δρ_min_ = −0.19 e Å^−^^3^
4 restraints	Extinction correction: *SHELXL2019/2* (Sheldrick, 2015b), Fc^*^=kFc[1+0.001xFc^2^λ^3^/sin(2θ)]^-1/4^
Primary atom site location: dual	Extinction coefficient: 0.0088 (13)

### 4-Amino-5-fluoro-1H-pyrimidin-2-one–4-hydroxybenzaldehyde (1/1) Special details

**Table:**

Geometry. All esds (except the esd in the dihedral angle between two l.s. planes) are estimated using the full covariance matrix. The cell esds are taken into account individually in the estimation of esds in distances, angles and torsion angles; correlations between esds in cell parameters are only used when they are defined by crystal symmetry. An approximate (isotropic) treatment of cell esds is used for estimating esds involving l.s. planes.
Refinement. The data collection, cell refinement, and data reduction were performed using CrysAlisPro (Rigaku OD, 2023). Structure solution was carried out with SHELXT 2014/5 (Sheldrick, 2015a) and refinement was done using SHELXL-2016/6 (Sheldrick, 2015b). Molecular graphics were prepared using PLATON (Spek, 2020), Mercury (Macrae *et al.*, 2020) and POVRay (Cason 2004).

### 4-Amino-5-fluoro-1H-pyrimidin-2-one–4-hydroxybenzaldehyde (1/1) Fractional atomic coordinates and isotropic or equivalent isotropic displacement parameters (Å^2^)

**Table:**

	*x*	*y*	*z*	*U*_iso_*/*U*_eq_	
F1A	0.6004 (2)	0.39753 (7)	0.63425 (3)	0.0557 (2)	
O1A	−0.1067 (2)	0.40145 (8)	0.79019 (3)	0.0439 (2)	
N1A	0.1299 (2)	0.27556 (9)	0.73165 (3)	0.0331 (2)	
N2A	0.1501 (2)	0.51932 (9)	0.73260 (3)	0.0344 (2)	
H1	0.076 (3)	0.5966 (14)	0.7444 (6)	0.050 (4)*	
N3A	0.3795 (3)	0.15570 (10)	0.67141 (4)	0.0401 (3)	
H1CC	0.492 (3)	0.1523 (16)	0.6446 (5)	0.049 (4)*	
H1A	0.294 (3)	0.0798 (14)	0.6845 (5)	0.045 (4)*	
C1A	0.4154 (3)	0.40215 (11)	0.67318 (4)	0.0351 (3)	
C2A	0.3072 (2)	0.27452 (11)	0.69223 (4)	0.0313 (2)	
C3A	0.0531 (2)	0.39737 (10)	0.75270 (4)	0.0323 (2)	
C4A	0.3331 (3)	0.52236 (11)	0.69305 (4)	0.0356 (3)	
H4A	0.399417	0.605918	0.680151	0.043*	
O1B	1.1638 (2)	0.27251 (10)	0.35919 (3)	0.0520 (3)	
H1B	1.074 (4)	0.2080 (18)	0.3414 (7)	0.076 (5)*	
O2B	0.6793 (3)	0.13585 (11)	0.57435 (3)	0.0605 (3)	
C1B	1.0816 (3)	0.25581 (12)	0.40653 (4)	0.0383 (3)	
C2B	0.8831 (3)	0.14938 (12)	0.42006 (4)	0.0421 (3)	
H2B	0.798860	0.087622	0.396053	0.050*	
C3B	0.8117 (3)	0.13552 (13)	0.46892 (4)	0.0450 (3)	
H3B	0.677170	0.064757	0.477749	0.054*	
C4B	0.9386 (3)	0.22637 (12)	0.50533 (4)	0.0401 (3)	
C5B	1.1361 (3)	0.33238 (13)	0.49140 (4)	0.0440 (3)	
H5B	1.222274	0.393527	0.515464	0.053*	
C6B	1.2064 (3)	0.34827 (13)	0.44238 (5)	0.0471 (3)	
H6B	1.336473	0.420430	0.433386	0.056*	
C7B	0.8656 (3)	0.21566 (14)	0.55746 (4)	0.0474 (3)	
H7B	0.972343	0.275878	0.579901	0.057*	

### 4-Amino-5-fluoro-1H-pyrimidin-2-one–4-hydroxybenzaldehyde (1/1) Atomic displacement parameters (Å^2^)

**Table:**

	*U*^11^	*U*^22^	*U*^33^	*U*^12^	*U*^13^	*U*^23^
F1A	0.0776 (5)	0.0414 (4)	0.0534 (5)	−0.0025 (3)	0.0416 (4)	0.0020 (3)
O1A	0.0648 (5)	0.0344 (4)	0.0352 (4)	0.0058 (4)	0.0229 (4)	0.0028 (3)
N1A	0.0453 (5)	0.0265 (4)	0.0287 (4)	−0.0013 (3)	0.0106 (4)	0.0013 (3)
N2A	0.0456 (5)	0.0250 (4)	0.0338 (5)	0.0007 (3)	0.0103 (4)	−0.0009 (3)
N3A	0.0588 (6)	0.0295 (5)	0.0341 (5)	−0.0013 (4)	0.0177 (4)	−0.0018 (4)
C1A	0.0421 (6)	0.0337 (6)	0.0310 (5)	−0.0024 (4)	0.0131 (4)	0.0025 (4)
C2A	0.0383 (5)	0.0296 (5)	0.0265 (5)	−0.0006 (4)	0.0053 (4)	0.0009 (4)
C3A	0.0416 (5)	0.0285 (5)	0.0274 (5)	0.0006 (4)	0.0065 (4)	0.0018 (4)
C4A	0.0423 (6)	0.0290 (5)	0.0364 (5)	−0.0035 (4)	0.0096 (4)	0.0047 (4)
O1B	0.0734 (6)	0.0513 (5)	0.0327 (4)	−0.0134 (4)	0.0137 (4)	0.0001 (4)
O2B	0.0838 (7)	0.0605 (6)	0.0404 (5)	0.0017 (5)	0.0255 (5)	0.0006 (4)
C1B	0.0477 (6)	0.0370 (5)	0.0311 (5)	0.0040 (4)	0.0082 (4)	0.0018 (4)
C2B	0.0536 (7)	0.0389 (6)	0.0343 (6)	−0.0031 (5)	0.0075 (5)	−0.0055 (4)
C3B	0.0549 (7)	0.0419 (6)	0.0397 (6)	−0.0053 (5)	0.0140 (5)	−0.0004 (5)
C4B	0.0464 (6)	0.0422 (6)	0.0324 (5)	0.0101 (5)	0.0080 (4)	−0.0005 (4)
C5B	0.0528 (7)	0.0425 (6)	0.0367 (6)	0.0017 (5)	0.0026 (5)	−0.0072 (5)
C6B	0.0595 (7)	0.0406 (6)	0.0418 (6)	−0.0090 (5)	0.0083 (5)	−0.0015 (5)
C7B	0.0569 (7)	0.0521 (7)	0.0342 (6)	0.0110 (6)	0.0102 (5)	−0.0031 (5)

### 4-Amino-5-fluoro-1H-pyrimidin-2-one–4-hydroxybenzaldehyde (1/1) Geometric parameters (Å, º)

**Table:**

F1A—C1A	1.3498 (12)	O1B—H1B	0.857 (15)
O1A—C3A	1.2521 (13)	O2B—C7B	1.2122 (17)
N1A—C2A	1.3397 (13)	C1B—C6B	1.3889 (17)
N1A—C3A	1.3558 (13)	C1B—C2B	1.3907 (16)
N2A—C4A	1.3578 (14)	C2B—C3B	1.3746 (16)
N2A—C3A	1.3715 (13)	C2B—H2B	0.9300
N2A—H1	0.877 (13)	C3B—C4B	1.3920 (17)
N3A—C2A	1.3231 (14)	C3B—H3B	0.9300
N3A—H1CC	0.891 (13)	C4B—C5B	1.3887 (18)
N3A—H1A	0.900 (12)	C4B—C7B	1.4594 (15)
C1A—C4A	1.3353 (15)	C5B—C6B	1.3791 (17)
C1A—C2A	1.4235 (14)	C5B—H5B	0.9300
C4A—H4A	0.9300	C6B—H6B	0.9300
O1B—C1B	1.3520 (13)	C7B—H7B	0.9300
			
C2A—N1A—C3A	120.00 (8)	O1B—C1B—C2B	122.30 (10)
C4A—N2A—C3A	121.95 (9)	C6B—C1B—C2B	119.97 (10)
C4A—N2A—H1	120.1 (10)	C3B—C2B—C1B	119.96 (11)
C3A—N2A—H1	117.8 (10)	C3B—C2B—H2B	120.0
C2A—N3A—H1CC	121.8 (10)	C1B—C2B—H2B	120.0
C2A—N3A—H1A	115.6 (9)	C2B—C3B—C4B	120.64 (11)
H1CC—N3A—H1A	122.4 (14)	C2B—C3B—H3B	119.7
C4A—C1A—F1A	121.33 (9)	C4B—C3B—H3B	119.7
C4A—C1A—C2A	120.77 (10)	C5B—C4B—C3B	118.92 (10)
F1A—C1A—C2A	117.90 (9)	C5B—C4B—C7B	118.91 (11)
N3A—C2A—N1A	119.97 (9)	C3B—C4B—C7B	122.16 (11)
N3A—C2A—C1A	120.74 (9)	C6B—C5B—C4B	120.93 (11)
N1A—C2A—C1A	119.29 (9)	C6B—C5B—H5B	119.5
O1A—C3A—N1A	121.43 (9)	C4B—C5B—H5B	119.5
O1A—C3A—N2A	118.86 (9)	C5B—C6B—C1B	119.58 (11)
N1A—C3A—N2A	119.71 (9)	C5B—C6B—H6B	120.2
C1A—C4A—N2A	118.21 (9)	C1B—C6B—H6B	120.2
C1A—C4A—H4A	120.9	O2B—C7B—C4B	126.30 (12)
N2A—C4A—H4A	120.9	O2B—C7B—H7B	116.9
C1B—O1B—H1B	107.7 (13)	C4B—C7B—H7B	116.9
O1B—C1B—C6B	117.73 (11)		
			
C3A—N1A—C2A—N3A	−179.36 (10)	O1B—C1B—C2B—C3B	178.79 (11)
C3A—N1A—C2A—C1A	0.55 (16)	C6B—C1B—C2B—C3B	−0.23 (19)
C4A—C1A—C2A—N3A	177.61 (11)	C1B—C2B—C3B—C4B	−0.64 (19)
F1A—C1A—C2A—N3A	−1.78 (16)	C2B—C3B—C4B—C5B	0.72 (18)
C4A—C1A—C2A—N1A	−2.30 (17)	C2B—C3B—C4B—C7B	179.39 (11)
F1A—C1A—C2A—N1A	178.31 (9)	C3B—C4B—C5B—C6B	0.07 (18)
C2A—N1A—C3A—O1A	−178.50 (10)	C7B—C4B—C5B—C6B	−178.65 (11)
C2A—N1A—C3A—N2A	1.84 (16)	C4B—C5B—C6B—C1B	−0.9 (2)
C4A—N2A—C3A—O1A	177.67 (10)	O1B—C1B—C6B—C5B	−178.06 (11)
C4A—N2A—C3A—N1A	−2.66 (16)	C2B—C1B—C6B—C5B	1.01 (19)
F1A—C1A—C4A—N2A	−179.10 (9)	C5B—C4B—C7B—O2B	173.98 (12)
C2A—C1A—C4A—N2A	1.53 (17)	C3B—C4B—C7B—O2B	−4.7 (2)
C3A—N2A—C4A—C1A	0.92 (17)		

### 4-Amino-5-fluoro-1H-pyrimidin-2-one–4-hydroxybenzaldehyde (1/1) Hydrogen-bond geometry (Å, º)

**Table:**

*D*—H···*A*	*D*—H	H···*A*	*D*···*A*	*D*—H···*A*
N2*A*—H1···N1*A*^i^	0.88 (1)	2.06 (1)	2.9354 (12)	175 (1)
N3*A*—H1*CC*···O2*B*	0.89 (1)	2.10 (1)	2.9848 (13)	170 (1)
N3*A*—H1*A*···O1*A*^ii^	0.90 (1)	2.04 (1)	2.9328 (12)	176 (1)
C4*A*—H4*A*···O1*B*^iii^	0.93	2.48	3.2905 (14)	145
O1*B*—H1*B*···O1*A*^iv^	0.86 (2)	1.85 (2)	2.6934 (13)	166 (2)
C6*B*—H6*B*···F1*A*^iii^	0.93	2.56	3.3446 (14)	143
C7*B*—H7*B*···F1*A*	0.93	2.51	2.9886 (14)	112

Symmetry codes: (i) −*x*, *y*+1/2, −*z*+3/2; (ii) −*x*, *y*−1/2, −*z*+3/2; (iii) −*x*+2, −*y*+1, −*z*+1; (iv) *x*+1, −*y*+1/2, *z*−1/2.
